# 2-(4-Bromo­phen­yl)-*N*-[3-(1*H*-imidazol-1-yl)prop­yl]quinazolin-4-amine

**DOI:** 10.1107/S1600536812028115

**Published:** 2012-06-30

**Authors:** Marcia Pérez-Fehrmann, Victor Kesternich, Felipe Verdugo, Philippe Christen, Céline Besnard

**Affiliations:** a Departamento de Química, Universidad Católica del Norte, Antofagasta, Chile; b School of Pharmaceutical Sciences, University of Geneva, Quai Ernest-Ansermet 30, CH-1211, Geneva 4, Switzerland; c Laboratoire de Cristallographie, University of Geneva, Quai Ernest-Ansermet 24, CH-1211 Geneva 4, Switzerland

## Abstract

In the title compound, C_20_H_18_BrN_5_, the bromo­phenyl-substituted quinazoline unit is essentially planar [maximum deviation = 0.098 (3) Å] and makes a dihedral angle of 56.04 (14)° with the imidazole ring. In the crystal, mol­ecules are associated by pairs of N—H⋯N hydrogen bonds to form inversion dimers. All the quinazoline planar systems are oriented almost perpendicular to the [110] direction, making π–π inter­actions possible between adjacent dimers [centroid–centroid distances = 3.7674 (16) and 3.7612 (17) Å]. There are also a number of C—H⋯π inter­actions present. The crystal is a nonmerohedral twin, with a minor twin fraction of 0.47.

## Related literature

For general background on the biological properties of imidazo quinazolines, see: Aguilar *et al.* (2002 ▶); Rohini *et al.* (2009 ▶). For imidazo quinazoline structures, see: Asproni *et al.* (2011 ▶); Connolly *et al.* (2005 ▶). For synthetic details, see: Okano *et al.* (2009 ▶).
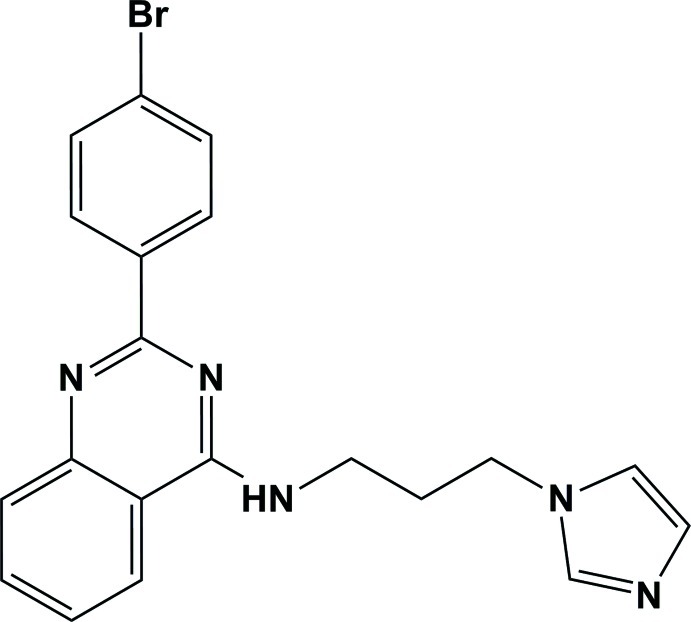


## Experimental

### 

#### Crystal data

C_20_H_18_BrN_5_
*M* *_r_* = 408.30
Triclinic, 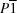

*a* = 8.8557 (7) Å
*b* = 9.5113 (6) Å
*c* = 11.3730 (7) Å
α = 99.682 (5)°
β = 101.432 (6)°
γ = 97.211 (6)°
*V* = 912.96 (11) Å^3^
*Z* = 2
Cu *K*α radiation
μ = 3.17 mm^−1^
*T* = 180 K
0.4 × 0.2 × 0.07 mm

#### Data collection

Agilent SuperNova, Dual, Cu, Atlas diffractometer
Absorption correction: multi-scan (*CrysAlis PRO*; Agilent, 2010 ▶) *T*_min_ = 0.343, *T*_max_ = 1.000
6845 measured reflections
6845 independent reflections
6259 reflections with *I* > 2σ(*I*)

#### Refinement

*R*[*F* ^2^ > 2σ(*F* ^2^)] = 0.073
*wR*(*F* ^2^) = 0.216
*S* = 1.12
6845 reflections
236 parameters
H-atom parameters constrained
Δρ_max_ = 0.94 e Å^−3^
Δρ_min_ = −0.87 e Å^−3^

### 

Data collection: *CrysAlis PRO* (Agilent, 2010 ▶); cell refinement: *CrysAlis PRO*; data reduction: *CrysAlis PRO*; program(s) used to solve structure: *SHELXS97* (Sheldrick, 2008 ▶); program(s) used to refine structure: *SHELXL97* (Sheldrick, 2008 ▶); molecular graphics: *OLEX2* (Dolomanov *et al.*, 2009 ▶); software used to prepare material for publication: *OLEX2* and *publCIF* (Westrip, 2010 ▶).

## Supplementary Material

Crystal structure: contains datablock(s) global, I. DOI: 10.1107/S1600536812028115/su2454sup1.cif

Structure factors: contains datablock(s) I. DOI: 10.1107/S1600536812028115/su2454Isup2.hkl

Supplementary material file. DOI: 10.1107/S1600536812028115/su2454Isup3.cml

Additional supplementary materials:  crystallographic information; 3D view; checkCIF report

## Figures and Tables

**Table 1:** Hydrogen-bond geometry (Å, °) *Cg*1 is the centroid of the N2,N5,C1–C3 ring; *Cg*3 is the centroid of the C11–C16 ring; *Cg*4 is the centroid of the C19–C24 ring.

*D*—H⋯*A*	*D*—H	H⋯*A*	*D*⋯*A*	*D*—H⋯*A*
N9—H9⋯N2^i^	0.86	2.14	2.949 (4)	156
C1—H1⋯*Cg*4^ii^	0.93	2.84	3.605 (3)	140
C4—H4⋯*Cg*3^iii^	0.93	2.78	3.509 (4)	136
C14—H14⋯*Cg*1^iv^	0.93	2.88	3.527 (3)	128

Symmetry codes: (i) 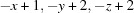; (ii) 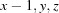; (iii) 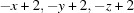; (iv) 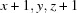.

**Fig. 1.**
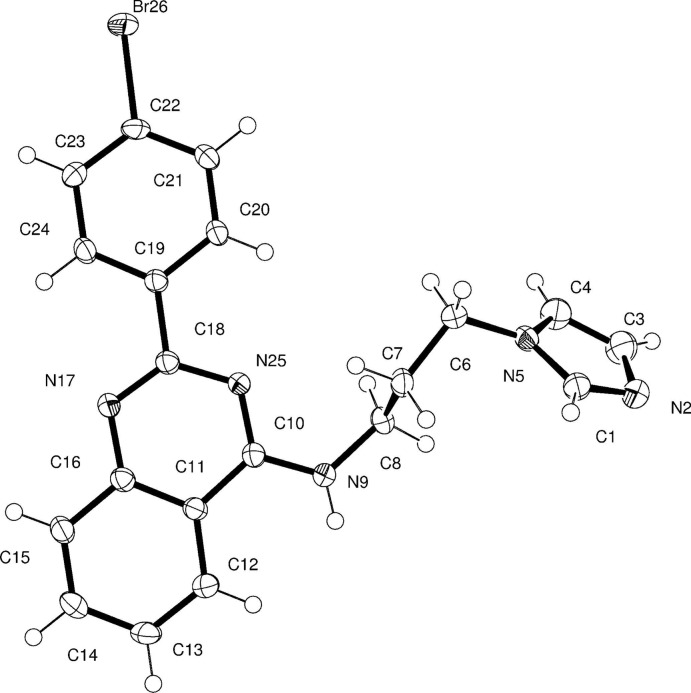
A view of the molecular structure of the title molecule with the atom numbering. The displacement ellipsoids are drawn at the 50% probability level.

**Fig. 2.**
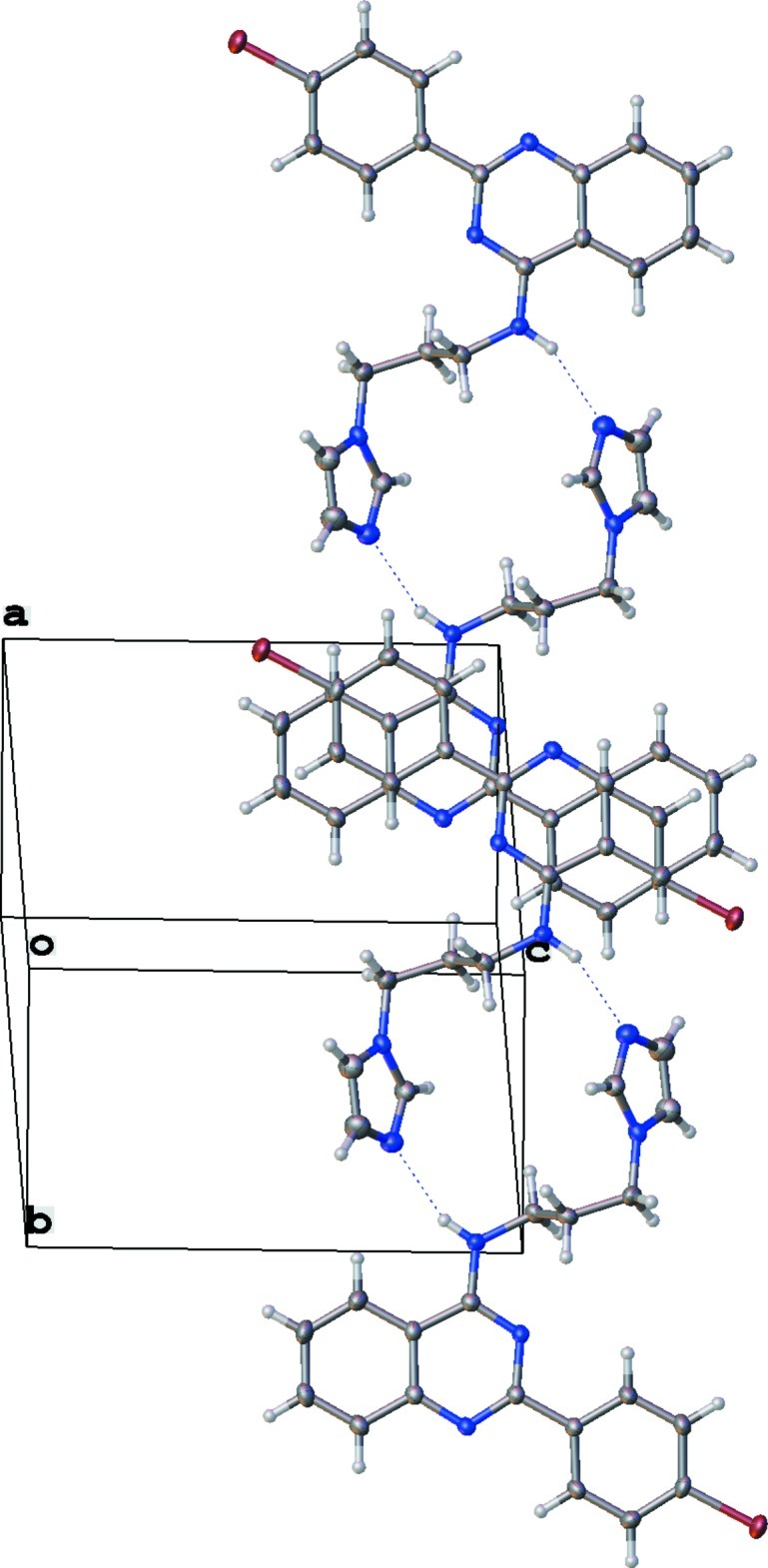
A partial view perpendicular to (110) of the crystal packing of the title compound, showing the N-H···N hydrogen bonded (blue dashed lines; Table 1) inversion dimers and the overlap of the inversion related bromophenyl substituted quinazoline units.

**Fig. 3.**
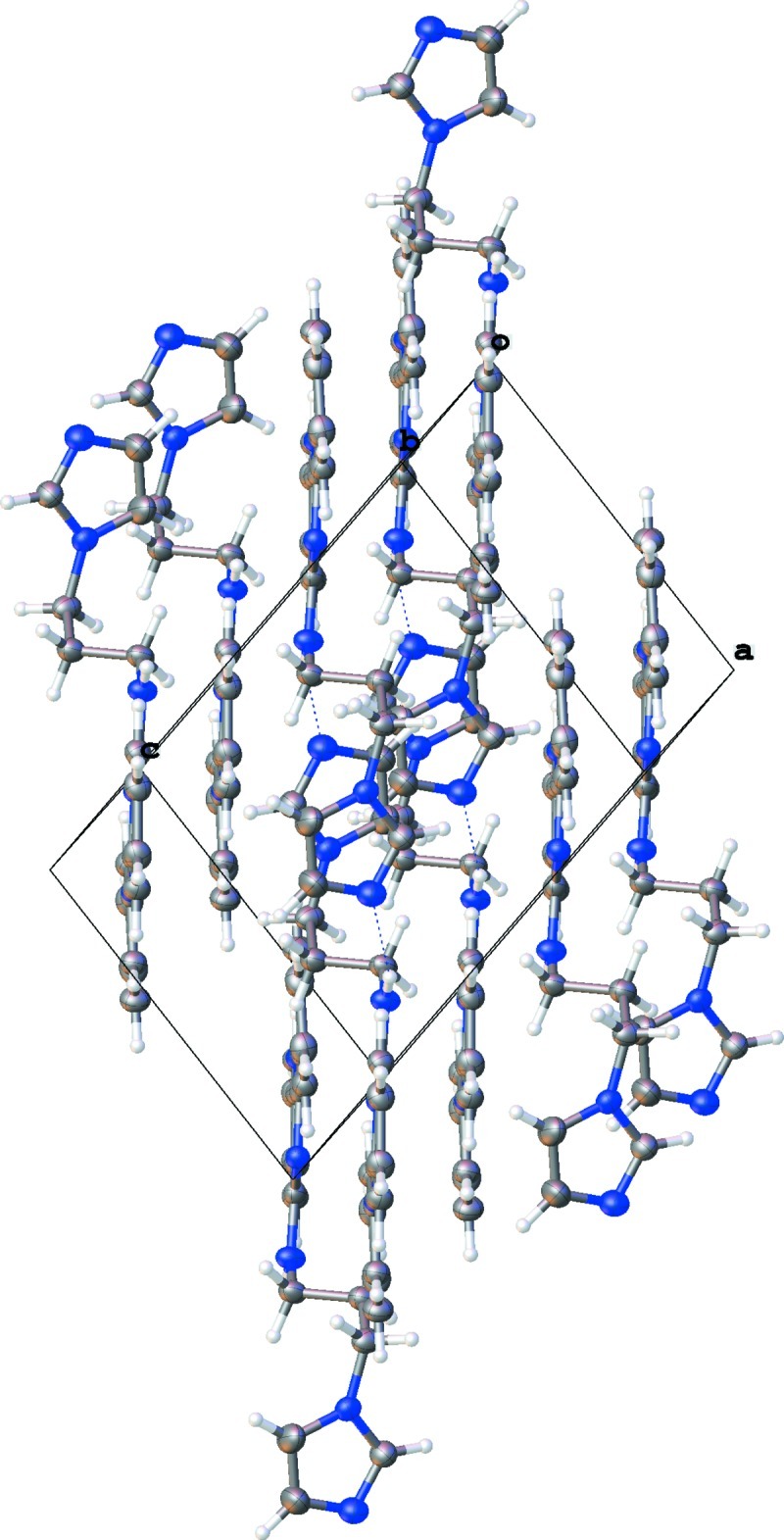
A view along the c axis of the crystal packing of the title compound. Hydrogen bonds are shown as blue dashed lines (see Table 1 for details).

## supplementary crystallographic information

### Comment

Imidazo quinazolines (Connolly *et al.*, 2005; Asproni *et al.*, 2011) are heterocyclic compounds that exhibit a wide range of biological activities such as, antibacterial, antifungal, and antitumor (Rohini *et al.*, 2009). The importance of this type of structure is linked to the fact that new drugs are permanently required owing to the fact that microorganisms are mutating continuously (Aguilar *et al.*, 2002).

In the title compound, Fig. 1, the bromophenyl substituted quinazoline unit is essentially planar, with a maximum deviation 0.098 (3)Å for atom C20, and makes a dihedral angle of 56.04 (14) ° with the imidazole ring.

In the crystal, the molecules are associated *via* N-H···N hydrogen bonds, involving the imidazole function (N2) and the NH group to form inversion dimers (Table 1 and Fig. 2). All the planar quinazoline systems are oriented almost perpendicular to direction [110] making π···π interactions possible between adjacent dimers [Fig 3; Cg2···Cg4^i^ 3.7674 (16) Å; Cg3···Cg4^i^ 3.7612 (17) Å; symmetry code (i) -x+2, -y+1, -z+2; Cg2, Cg3 and Cg4 are the centroids of rings (N25,C10,C11,C16,N17,C18), (C11-C16) and (C19-C20), respectively]. There are also a number of C-H···π interactions present (Table 1).

### Experimental

Both the starting reagent, 4-chloro-2-(4-bromophenyl)quinazoline, and the title compound were synthesized as described by (Okano *et al.*, 2009). To a stirred solution of 0.4 g of 4-chloro-2-(4-bromophenyl)quinazoline (1,3 mmol) in 6 ml of *N*,*N*-dimethylformamide, a mixture of 0.4 ml of Et_3_N (2.6 mmol) and 0.2 ml of 1-(3-aminopropyl)-imidazole (1.6 mmol) were added. The reaction mixture was stirred at room temperature for 4 h. After completion of the reaction 30 ml of cold water were added giving a white precipitate of the title compound that was purified in acetone [Yield 95%]. Recrystallization in acetone at room temperature afforded colourless plate-like crystals suitable for X-ray diffraction analysis (M.p. 438-440 K).

### Refinement

The crystal is a non-merohedral twin, with a minor twin fraction of 0.47. Two components rotated by 180 ° around an axis close to the *a* axis were used to produce an HKLF5 file that was used in the refinement. The H atoms were included in calculated positions and treated as riding atoms: N-H = 0.86 Å, C-H = 0.93 and 0.97 Å for CH and CH_2_ H atoms, respectively, with U_iso_(H) = 1.2U_eq_(parent N or C atom).

### Crystal data

**Table:**

C_20_H_18_BrN_5_	*Z* = 2
*M**_r_* = 408.30	*F*(000) = 416
Triclinic, *P*1	*D*_x_ = 1.485 Mg m^−^^3^
Hall symbol: -P 1	Melting point = 438–440 K
*a* = 8.8557 (7) Å	Cu *K*α radiation, λ = 1.5418 Å
*b* = 9.5113 (6) Å	Cell parameters from 4656 reflections
*c* = 11.3730 (7) Å	θ = 4.0–72.6°
α = 99.682 (5)°	µ = 3.17 mm^−^^1^
β = 101.432 (6)°	*T* = 180 K
γ = 97.211 (6)°	Plate, colourless
*V* = 912.96 (11) Å^3^	0.4 × 0.2 × 0.07 mm

### Data collection

**Table:**

Agilent SuperNova, Dual, Cu, Atlas diffractometer	6845 independent reflections
Radiation source: SuperNova (Cu) X-ray Source	6259 reflections with *I* > 2σ(*I*)
Mirror monochromator	*R*_int_ = 0.000
Detector resolution: 10.4679 pixels mm^-1^	θ_max_ = 73.4°, θ_min_ = 4.1°
ω scans	*h* = −10→10
Absorption correction: multi-scan (*CrysAlis PRO*; Agilent, 2010)	*k* = −11→11
*T*_min_ = 0.343, *T*_max_ = 1.000	*l* = −14→14
6845 measured reflections	

### Refinement

**Table:**

Refinement on *F*^2^	Primary atom site location: structure-invariant direct methods
Least-squares matrix: full	Secondary atom site location: difference Fourier map
*R*[*F*^2^ > 2σ(*F*^2^)] = 0.073	Hydrogen site location: inferred from neighbouring sites
*wR*(*F*^2^) = 0.216	H-atom parameters constrained
*S* = 1.12	*w* = 1/[σ^2^(*F*_o_^2^) + (0.1707*P*)^2^ + 0.1234*P*] where *P* = (*F*_o_^2^ + 2*F*_c_^2^)/3
6845 reflections	(Δ/σ)_max_ = 0.001
236 parameters	Δρ_max_ = 0.94 e Å^−^^3^
0 restraints	Δρ_min_ = −0.87 e Å^−^^3^

### Special details

**Table:**

Geometry. Bond distances, angles etc. have been calculated using the rounded fractional coordinates. All su's are estimated from the variances of the (full) variance-covariance matrix. The cell esds are taken into account in the estimation of distances, angles and torsion angles
Refinement. The crystal is twinned. Two components rotated by 180 degrees around an axis close to the *a* axis were used to produce an hklf5 file that was used in the refinement. The twin fraction is 0.47.

### Fractional atomic coordinates and isotropic or equivalent isotropic displacement parameters (Å^2^)

**Table:**

	*x*	*y*	*z*	*U*_iso_*/*U*_eq_	
Br26	1.29926 (4)	0.38228 (3)	0.53762 (3)	0.0344 (2)	
N2	0.3265 (3)	0.9940 (3)	0.7534 (3)	0.0332 (8)	
N5	0.5204 (3)	0.8708 (2)	0.7429 (2)	0.0226 (6)	
N9	0.8641 (3)	0.8764 (3)	1.0859 (2)	0.0249 (7)	
N17	1.2170 (3)	0.6321 (2)	1.1230 (2)	0.0219 (6)	
N25	1.0223 (2)	0.7362 (2)	1.0039 (2)	0.0208 (6)	
C1	0.3882 (3)	0.8826 (3)	0.7826 (3)	0.0271 (8)	
C3	0.4247 (4)	1.0562 (3)	0.6895 (3)	0.0363 (10)	
C4	0.5446 (4)	0.9818 (3)	0.6823 (3)	0.0320 (9)	
C6	0.6173 (3)	0.7581 (3)	0.7585 (3)	0.0281 (8)	
C7	0.6775 (3)	0.7528 (3)	0.8922 (3)	0.0244 (8)	
C8	0.7867 (3)	0.8907 (3)	0.9637 (3)	0.0243 (8)	
C10	0.9798 (3)	0.7984 (3)	1.1023 (2)	0.0211 (7)	
C11	1.0576 (3)	0.7841 (3)	1.2228 (2)	0.0202 (7)	
C12	1.0225 (3)	0.8494 (3)	1.3326 (3)	0.0272 (8)	
C13	1.1050 (4)	0.8314 (3)	1.4436 (3)	0.0300 (8)	
C14	1.2239 (3)	0.7465 (3)	1.4478 (3)	0.0276 (8)	
C15	1.2587 (3)	0.6803 (3)	1.3418 (3)	0.0256 (8)	
C16	1.1774 (3)	0.6981 (3)	1.2261 (3)	0.0210 (7)	
C18	1.1381 (3)	0.6553 (3)	1.0193 (2)	0.0201 (7)	
C19	1.1755 (3)	0.5861 (3)	0.9036 (2)	0.0198 (7)	
C20	1.0851 (3)	0.5960 (3)	0.7895 (3)	0.0243 (7)	
C21	1.1213 (3)	0.5346 (3)	0.6819 (3)	0.0259 (8)	
C22	1.2489 (3)	0.4630 (3)	0.6873 (3)	0.0240 (8)	
C23	1.3403 (3)	0.4505 (3)	0.7974 (3)	0.0258 (8)	
C24	1.3025 (3)	0.5117 (3)	0.9049 (3)	0.0231 (7)	
H1	0.34540	0.81910	0.82590	0.0320*	
H3	0.41070	1.13760	0.65600	0.0440*	
H4	0.62630	1.00180	0.64420	0.0380*	
H6A	0.70550	0.77570	0.72110	0.0340*	
H6B	0.55640	0.66490	0.71630	0.0340*	
H7A	0.73260	0.67130	0.89680	0.0290*	
H7B	0.58930	0.73740	0.93000	0.0290*	
H8A	0.86520	0.91490	0.91860	0.0290*	
H8B	0.72720	0.96950	0.97080	0.0290*	
H9	0.83500	0.91890	1.14850	0.0300*	
H12	0.94310	0.90500	1.33010	0.0330*	
H13	1.08190	0.87550	1.51590	0.0360*	
H14	1.27960	0.73500	1.52300	0.0330*	
H15	1.33660	0.62310	1.34600	0.0310*	
H20	1.00000	0.64450	0.78660	0.0290*	
H21	1.06090	0.54110	0.60670	0.0310*	
H23	1.42530	0.40190	0.79930	0.0310*	
H24	1.36270	0.50330	0.97950	0.0280*	

### Atomic displacement parameters (Å^2^)

**Table:**

	*U*^11^	*U*^22^	*U*^33^	*U*^12^	*U*^13^	*U*^23^
Br26	0.0438 (3)	0.0463 (3)	0.0199 (2)	0.0198 (2)	0.0142 (2)	0.0076 (2)
N2	0.0312 (12)	0.0421 (14)	0.0294 (13)	0.0157 (11)	0.0077 (11)	0.0076 (12)
N5	0.0226 (10)	0.0276 (11)	0.0187 (11)	0.0058 (8)	0.0035 (9)	0.0079 (9)
N9	0.0255 (11)	0.0311 (12)	0.0193 (11)	0.0132 (9)	0.0041 (9)	0.0035 (9)
N17	0.0201 (10)	0.0284 (11)	0.0188 (11)	0.0081 (8)	0.0040 (8)	0.0066 (9)
N25	0.0192 (9)	0.0281 (11)	0.0164 (11)	0.0075 (8)	0.0041 (8)	0.0049 (9)
C1	0.0240 (12)	0.0355 (14)	0.0251 (14)	0.0070 (11)	0.0086 (11)	0.0102 (12)
C3	0.0420 (17)	0.0329 (15)	0.0386 (19)	0.0120 (12)	0.0108 (14)	0.0131 (14)
C4	0.0318 (14)	0.0332 (15)	0.0347 (17)	0.0042 (11)	0.0114 (12)	0.0131 (13)
C6	0.0297 (13)	0.0333 (14)	0.0211 (14)	0.0133 (11)	0.0043 (11)	0.0009 (11)
C7	0.0221 (12)	0.0281 (13)	0.0257 (14)	0.0093 (10)	0.0046 (10)	0.0101 (11)
C8	0.0269 (13)	0.0260 (13)	0.0219 (14)	0.0088 (10)	0.0031 (11)	0.0092 (11)
C10	0.0186 (11)	0.0244 (12)	0.0202 (13)	0.0044 (9)	0.0034 (10)	0.0049 (10)
C11	0.0190 (11)	0.0244 (12)	0.0168 (13)	0.0024 (9)	0.0039 (9)	0.0038 (10)
C12	0.0254 (12)	0.0342 (15)	0.0213 (14)	0.0093 (11)	0.0052 (11)	0.0008 (12)
C13	0.0322 (14)	0.0384 (15)	0.0175 (14)	0.0070 (12)	0.0060 (11)	−0.0014 (12)
C14	0.0289 (13)	0.0334 (14)	0.0180 (13)	0.0033 (11)	−0.0001 (11)	0.0055 (11)
C15	0.0231 (12)	0.0328 (14)	0.0224 (14)	0.0071 (10)	0.0031 (10)	0.0102 (12)
C16	0.0197 (11)	0.0243 (12)	0.0195 (13)	0.0024 (9)	0.0050 (10)	0.0063 (10)
C18	0.0187 (11)	0.0244 (12)	0.0185 (13)	0.0036 (9)	0.0053 (10)	0.0063 (10)
C19	0.0183 (11)	0.0250 (12)	0.0177 (13)	0.0045 (9)	0.0054 (9)	0.0066 (10)
C20	0.0199 (11)	0.0351 (14)	0.0190 (13)	0.0092 (10)	0.0023 (10)	0.0072 (11)
C21	0.0253 (12)	0.0358 (14)	0.0172 (13)	0.0085 (11)	0.0024 (10)	0.0072 (11)
C22	0.0283 (13)	0.0289 (13)	0.0163 (13)	0.0051 (10)	0.0097 (10)	0.0029 (11)
C23	0.0264 (12)	0.0311 (13)	0.0241 (14)	0.0127 (10)	0.0095 (11)	0.0068 (11)
C24	0.0217 (12)	0.0308 (13)	0.0197 (13)	0.0081 (10)	0.0041 (10)	0.0108 (11)

### Geometric parameters (Å, º)

**Table:**

Br26—C22	1.906 (3)	C19—C24	1.401 (4)
N2—C1	1.313 (4)	C19—C20	1.408 (4)
N2—C3	1.379 (4)	C20—C21	1.380 (4)
N5—C1	1.346 (4)	C21—C22	1.387 (4)
N5—C4	1.372 (4)	C22—C23	1.382 (4)
N5—C6	1.467 (4)	C23—C24	1.385 (4)
N9—C8	1.459 (4)	C1—H1	0.9300
N9—C10	1.340 (4)	C3—H3	0.9300
N17—C16	1.365 (4)	C4—H4	0.9300
N17—C18	1.314 (3)	C6—H6A	0.9700
N25—C10	1.321 (3)	C6—H6B	0.9700
N25—C18	1.359 (3)	C7—H7A	0.9700
N9—H9	0.8600	C7—H7B	0.9700
C3—C4	1.356 (5)	C8—H8A	0.9700
C6—C7	1.520 (5)	C8—H8B	0.9700
C7—C8	1.526 (4)	C12—H12	0.9300
C10—C11	1.443 (3)	C13—H13	0.9300
C11—C16	1.417 (4)	C14—H14	0.9300
C11—C12	1.408 (4)	C15—H15	0.9300
C12—C13	1.377 (5)	C20—H20	0.9300
C13—C14	1.403 (4)	C21—H21	0.9300
C14—C15	1.373 (4)	C23—H23	0.9300
C15—C16	1.417 (5)	C24—H24	0.9300
C18—C19	1.487 (3)		
			
C1—N2—C3	104.7 (3)	C22—C23—C24	118.6 (3)
C1—N5—C4	106.9 (2)	C19—C24—C23	121.4 (3)
C1—N5—C6	126.4 (2)	N2—C1—H1	124.00
C4—N5—C6	126.7 (3)	N5—C1—H1	124.00
C8—N9—C10	121.2 (2)	N2—C3—H3	125.00
C16—N17—C18	115.5 (2)	C4—C3—H3	125.00
C10—N25—C18	118.1 (2)	N5—C4—H4	127.00
C8—N9—H9	119.00	C3—C4—H4	127.00
C10—N9—H9	119.00	N5—C6—H6A	109.00
N2—C1—N5	112.3 (3)	N5—C6—H6B	109.00
N2—C3—C4	110.4 (3)	C7—C6—H6A	109.00
N5—C4—C3	105.7 (3)	C7—C6—H6B	109.00
N5—C6—C7	112.6 (2)	H6A—C6—H6B	108.00
C6—C7—C8	112.8 (2)	C6—C7—H7A	109.00
N9—C8—C7	112.5 (2)	C6—C7—H7B	109.00
N9—C10—N25	117.6 (2)	C8—C7—H7A	109.00
N9—C10—C11	121.7 (2)	C8—C7—H7B	109.00
N25—C10—C11	120.8 (2)	H7A—C7—H7B	108.00
C10—C11—C12	124.6 (3)	N9—C8—H8A	109.00
C12—C11—C16	120.0 (2)	N9—C8—H8B	109.00
C10—C11—C16	115.4 (2)	C7—C8—H8A	109.00
C11—C12—C13	120.4 (3)	C7—C8—H8B	109.00
C12—C13—C14	120.0 (3)	H8A—C8—H8B	108.00
C13—C14—C15	120.6 (3)	C11—C12—H12	120.00
C14—C15—C16	120.8 (3)	C13—C12—H12	120.00
N17—C16—C11	122.8 (3)	C12—C13—H13	120.00
C11—C16—C15	118.2 (3)	C14—C13—H13	120.00
N17—C16—C15	119.0 (3)	C13—C14—H14	120.00
N17—C18—C19	118.2 (2)	C15—C14—H14	120.00
N25—C18—C19	114.5 (2)	C14—C15—H15	120.00
N17—C18—N25	127.4 (2)	C16—C15—H15	120.00
C20—C19—C24	118.3 (2)	C19—C20—H20	120.00
C18—C19—C20	120.6 (2)	C21—C20—H20	120.00
C18—C19—C24	121.2 (2)	C20—C21—H21	120.00
C19—C20—C21	120.8 (3)	C22—C21—H21	120.00
C20—C21—C22	119.1 (3)	C22—C23—H23	121.00
Br26—C22—C23	119.7 (2)	C24—C23—H23	121.00
Br26—C22—C21	118.4 (2)	C19—C24—H24	119.00
C21—C22—C23	121.9 (3)	C23—C24—H24	119.00
			
C3—N2—C1—N5	0.8 (4)	C10—C11—C12—C13	−178.9 (3)
C1—N2—C3—C4	−0.5 (4)	C16—C11—C12—C13	0.6 (4)
C4—N5—C1—N2	−0.8 (4)	C10—C11—C16—N17	−0.4 (4)
C6—N5—C1—N2	−179.2 (3)	C10—C11—C16—C15	179.8 (3)
C1—N5—C4—C3	0.4 (3)	C12—C11—C16—N17	−180.0 (3)
C6—N5—C4—C3	178.8 (3)	C12—C11—C16—C15	0.2 (4)
C1—N5—C6—C7	−58.8 (4)	C11—C12—C13—C14	−0.6 (5)
C4—N5—C6—C7	123.2 (3)	C12—C13—C14—C15	−0.3 (5)
C10—N9—C8—C7	73.6 (3)	C13—C14—C15—C16	1.1 (4)
C8—N9—C10—N25	0.7 (4)	C14—C15—C16—N17	179.1 (3)
C8—N9—C10—C11	179.7 (3)	C14—C15—C16—C11	−1.0 (4)
C18—N17—C16—C11	1.1 (4)	N17—C18—C19—C20	174.0 (3)
C18—N17—C16—C15	−179.0 (3)	N17—C18—C19—C24	−7.1 (4)
C16—N17—C18—N25	−0.4 (4)	N25—C18—C19—C20	−5.5 (4)
C16—N17—C18—C19	−179.9 (2)	N25—C18—C19—C24	173.4 (3)
C18—N25—C10—N9	−179.2 (3)	C18—C19—C20—C21	178.6 (3)
C18—N25—C10—C11	1.8 (4)	C24—C19—C20—C21	−0.3 (4)
C10—N25—C18—N17	−1.1 (4)	C18—C19—C24—C23	−178.2 (3)
C10—N25—C18—C19	178.4 (2)	C20—C19—C24—C23	0.7 (4)
N2—C3—C4—N5	0.1 (4)	C19—C20—C21—C22	−0.3 (4)
N5—C6—C7—C8	−63.8 (3)	C20—C21—C22—Br26	−178.8 (2)
C6—C7—C8—N9	−170.6 (2)	C20—C21—C22—C23	0.5 (4)
N9—C10—C11—C12	−0.6 (5)	Br26—C22—C23—C24	179.2 (2)
N9—C10—C11—C16	179.9 (3)	C21—C22—C23—C24	−0.1 (4)
N25—C10—C11—C12	178.4 (3)	C22—C23—C24—C19	−0.5 (4)
N25—C10—C11—C16	−1.2 (4)		

### Hydrogen-bond geometry (Å, º)

Cg1 is the centroid of the N2,N5,C1–C3 ring; Cg3 is the centroid of the C11–C16 ring; Cg4 is the centroid of the C19–C24 ring.

**Table:**

*D*—H···*A*	*D*—H	H···*A*	*D*···*A*	*D*—H···*A*
N9—H9···N2^i^	0.86	2.14	2.949 (4)	156
C1—H1···*Cg*4^ii^	0.93	2.84	3.605 (3)	140
C4—H4···*Cg*3^iii^	0.93	2.78	3.509 (4)	136
C14—H14···*Cg*1^iv^	0.93	2.88	3.527 (3)	128

Symmetry codes: (i) −*x*+1, −*y*+2, −*z*+2; (ii) *x*−1, *y*, *z*; (iii) −*x*+2, −*y*+2, −*z*+2; (iv) *x*+1, *y*, *z*+1.
